# Macrophage Extracellular Traps Modulate the Compensatory Anti-inflammatory Response Syndrome through IL-33/ST2 Signaling in Severe Acute Pancreatitis

**DOI:** 10.7150/ijbs.116740

**Published:** 2025-09-22

**Authors:** Quan Zhou, Zhao Shi, Xuan Wang, Lingxi Meng, Kun Liu, Siqi Zhou, Yihang Jiang, Shuang Nie, Yuanyuan Yu, Hao Zhu, Mingdong Liu, Bo Kong, Helmut Friess, Lei Wang, Hongzhen Li, Shanshan Shen, Xiaoping Zou

**Affiliations:** 1 Department of Gastroenterology, Nanjing Drum Tower Hospital, Affiliated Hospital of Medical School, Nanjing University, Nanjing, China.; 2 Department of Gastroenterology, Nanjing Drum Tower Hospital, Clinical College of Nanjing Medical University, Nanjing, Jiangsu Province, China.; 3 Department of Gastroenterology, Affiliated Taikang Xianlin Drum Tower Hospital, Medical school of Nanjing University, Nanjing, Jiangsu Province, China.; 4 Department of Gastroenterology, Nanjing Drum Tower Hospital Clinical College of Nanjing University of Chinese Medicine, Nanjing, Jiangsu Province, China.; 5 Department of General, Visceral and Transplantation Surgery, University of Heidelberg, Heidelberg, Germany.; 6 Department of Surgery, Klinikum rechts der Isar, School of Medicine, Technical University of Munich, Munich, Germany.

**Keywords:** severe acute pancreatitis, macrophage extracellular traps, CARS, IL-33

## Abstract

Severe acute pancreatitis (SAP) is characterized by biphasic systemic inflammation, progressing from initial pro-inflammatory systemic inflammatory response syndrome (SIRS) to subsequent immunosuppressive compensatory anti-inflammatory response syndrome (CARS), which increases infection risks and predicts poor prognosis. Using a pancreatic duct ligation and caerulein-induced SAP murine model, we demonstrate that macrophage extracellular traps (METs) play a pivotal role in immune regulation. Mechanistically, acinar cell activation of the cGAS-STING pathway triggers pyroptosis-mediated IL-33 release. METs subsequently process IL-33 into highly bioactive isoforms through METs-derived proteases (including MMP-12), thereby initiating ST2 receptor-mediated type-2 immune responses. Clinical validation revealed elevated serum METs marker and IL-33 levels in SAP patients. Therapeutic interventions with DNase I and Cl-amidine significantly attenuated IL-33 release, Th2 cell activation, and disease severity in experimental models. Our findings establish METs as critical regulators of SAP-associated CARS and propose METs inhibition as a promising therapeutic strategy for SAP management.

## Introduction

Severe acute pancreatitis (SAP), the most critical form of acute pancreatitis, is associated with widespread organ dysfunction and elevated mortality 1. SAP progresses from localized pancreatic and peripancreatic inflammation to systemic inflammation, a condition known as systemic inflammatory response syndrome (SIRS), which ultimately leads to multiorgan failure. Subsequently, a phase of suppressed inflammatory response arises, termed compensatory anti-inflammatory response syndrome (CARS), emerges, rendering patients more susceptible to infections 2. Recent study has revealed that SIRS and CARS can occur in parallel, with alterations in their balance contributing to the complex immune status observed during the progression of SAP 3. However, the regulatory mechanisms underlying the balance between SIRS and CARS remain unclear, thereby hindering our understanding of the inflammatory state and disease severity as SAP progresses.

Both the innate and adaptive immune systems contribute to the modulation of inflammatory responses during the progression of SAP. Macrophages are the dominant immune cell population during pancreatitis, exhibiting high plasticity and playing a crucial role in both the innate and adaptive immune responses^4^. Infiltrating macrophages mediate proinflammatory responses while also activating Th2 cell-mediated immunosuppressive reactions in mouse models 3. This dual functionality suggests that macrophages serve as a bridge between the innate and adaptive immune systems in pancreatitis, regulating the balance between SIRS and CARS. Extracellular traps (ETs) are extracellular DNA fiber structures containing granule proteins, produced by neutrophils, macrophages, eosinophils, and other cells. These structures are associated with the progression of various inflammatory diseases 5, 6. Despite the extensive research focus on the functions of neutrophil extracellular traps (NETs) in inflammation diseases, the role of macrophage extracellular traps (METs) in pancreatitis have received little attention. Given that macrophages are the dominant cell lineage in the injured pancreas and that ETs have been reported to regulate T-cell differentiation, we hypothesize that METs may mediate immune dysfunction in SAP by modulating adaptive immunity.

In this study, we employed an established mouse model of SAP, induced through a combination of pancreatic duct ligation and caerulein administration, to explore the role of METs in this condition. We demonstrated that during SAP, METs interact with acinar cells, activating the cGAS-STING-GSDMD signaling pathway and leading to the release of IL-33 into the extracellular space. The proteases carried by METs then cleave IL-33, enhancing its bioactivity. This cleaved IL-33 binds to its receptor ST2, promoting the differentiation of T cells into Th2 cells, which subsequently mediate CARS during SAP. Our findings suggest that clearance of METs or the inhibition of their formation represent a potential therapeutic strategy to mitigate excessive immune suppression in SAP, thereby reducing the risk of multi-organ failure and secondary infections.

## Results

### Formation of METs during SAP Progression

**Figure 1:**
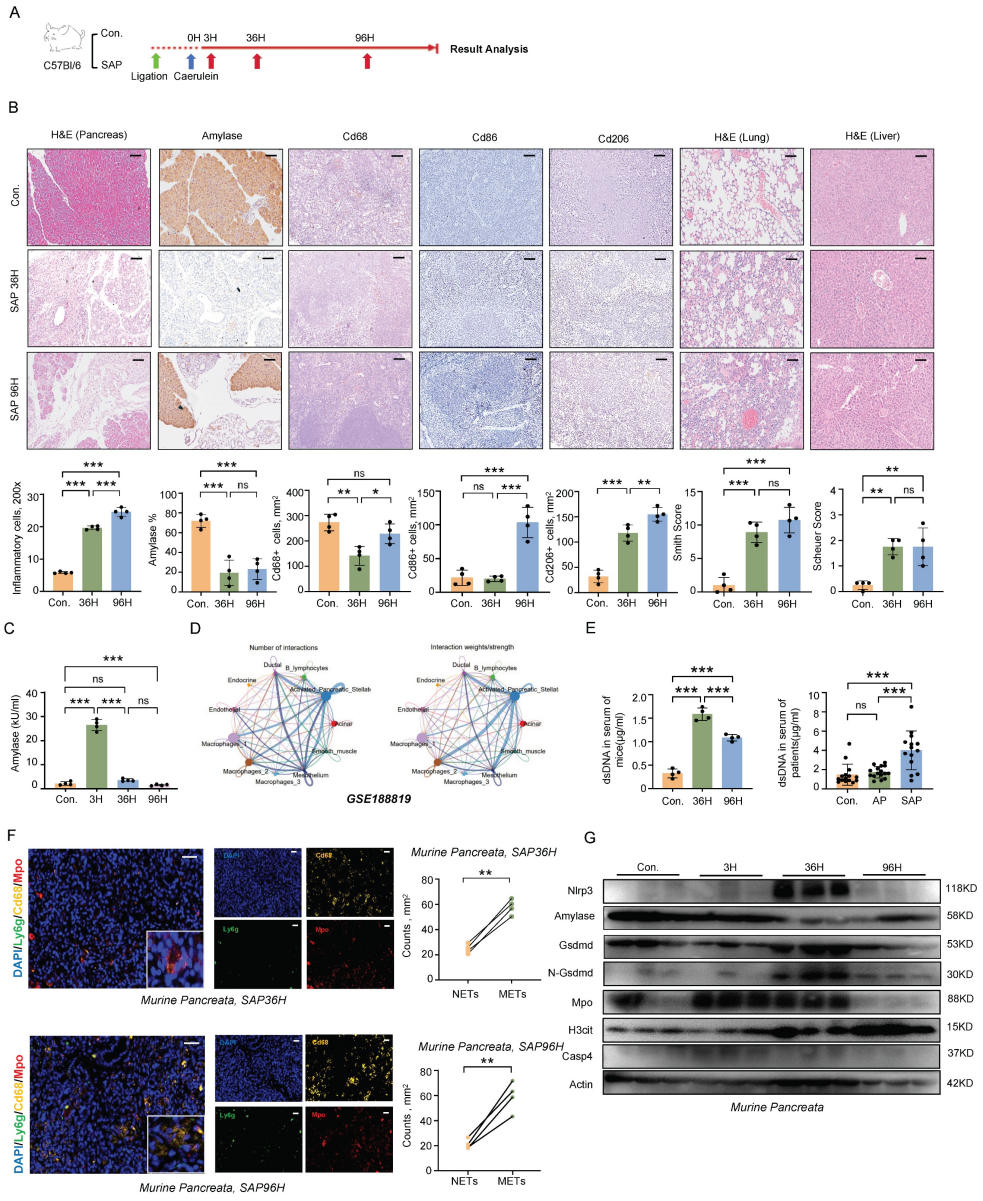
**Formation of METs during SAP progression. A)** Schematic diagram of the establishment method for the SAP mouse model. **B)** Representative images and quantitative assessments depict pancreatic inflammation cell infiltration, amylase staining of pancreata, splenic immunostaining for Cd68^+^, Cd86^+^, and Cd206^+^ cells, as well as histological scoring of lung and liver tissues in control and SAP mice (n=4 mice per group). Scale bar=50μm. **C)** Serum amylase levels from control and SAP mice (n=4 mice per group). **D)** Single-cell transcriptomic analysis of acute pancreatitis using CellChat. **E)** dsDNA levels in serum samples from control and SAP mice (left panel, with n=4 mice per group), and dsDNA levels in serum from control individuals, patients with acute pancreatitis, and those with SAP (right panel, with n=14~16 subjects per group). **F)** Representative immunofluorescence images of Mpo (red), Cd68 (gold) and Ly6g (green) in pancreatic tissues from SAP mice at 36 hours and 96 hours post-modeling (left panel), and quantitative assessment of METs and NETs counts in pancreatic tissues at both time points (right panel). Scale bar=50μm. **G)** Western blot analysis of Nlrp3, Amylase, Gsdmd, N-Gsdmd, Mpo, H3cit and Casp4 in the pancreata of control and SAP mice at all time points (n=3 mice per group). Statistical significance for **B, C** and **E** was determined using a one-way ANOVA with multiple comparisons test, and **F** was assessed using paired Student's t-tests. Data are presented as mean ±SD. Statistical significance: *P < 0.05, **P < 0.01, ***P < 0.001, ns. not significant. SAP, severe acute pancreatitis; NETs, neutrophil extracellular traps; METs, macrophage extracellular traps.

A murine model of SAP was induced in C57BL/6 wild-type mice by combining pancreatic duct ligation with caerulein stimulation (Figure 1A). This induction triggered a cascade of pathological changes, including necrosis of pancreatic acinar cells, infiltration of inflammatory cells, damage to lung and liver tissues, and elevated serum amylase levels 7 (Figure 1B, C). In the course of SAP, macrophages emerged as the most abundant immune cells. During the progression of SAP, we observed that both M1 and M2 macrophages were upregulated in the spleen (Figure 1B). Notably, macrophages in pancreatitis interact with various cell types to mediate complex inflammatory responses, thereby further complicating the disease process (Figure 1D).

Macrophages, similar to neutrophils, have the capacity to produce ETs, which are composed of extracellular chromatin DNA fibers impregnated with granule proteins. Despite their potential significance, the role of METs in SAP has received limited attention. Here, we observed a significant increase in the serum levels of double-stranded DNA (dsDNA), a marker for ETs, during SAP in both mice and patients (Figure 1E). Notably, within the injured pancreas of mice, METs were found to be more predominant compared to NETs (Figure 1F). Furthermore, gasdermin D (GSDMD)-dependent pyroptosis has been identified as an inducer of ETs formation 8. In the pancreas of mice, we found that pyroptosis occurs concurrently with ETs formation, suggesting a potential correlation between these two processes during SAP (Figure 1G). These findings indicate that METs are formed during the progression of SAP and may contribute to the regulation of inflammatory responses.

### METs Clearance Alleviates the Severity of SAP in Mice

**Figure 2:**
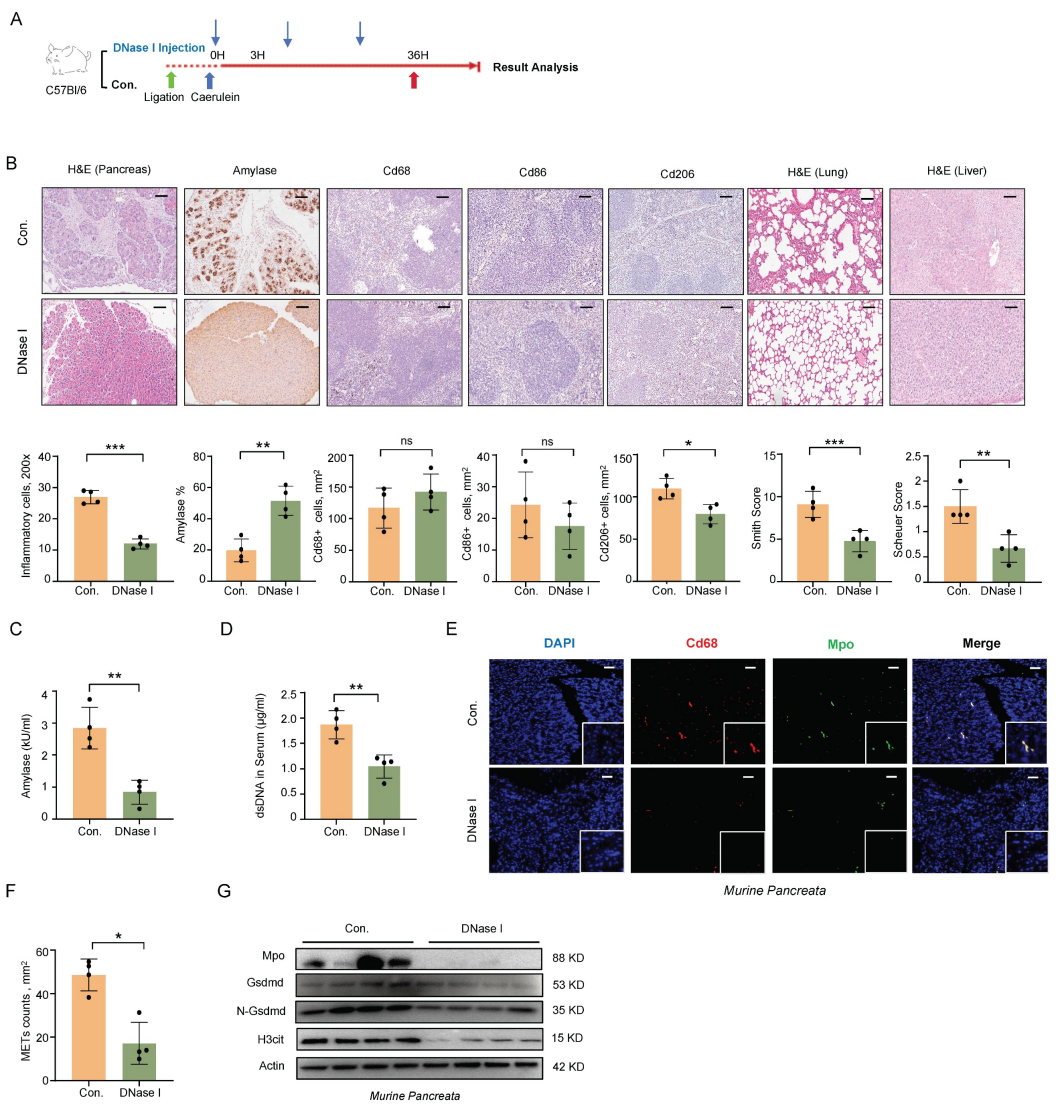
** METs clearance alleviates the severity of SAP in mice. A)** After SAP induction, DNase I was injected every 12 hours post-modeling at 1 mg/kg. **B)** Representative images and quantitative analysis illustrate pancreatic inflammation cell infiltration, amylase staining of pancreata, splenic immunostaining for Cd68^+^, Cd86^+^, and Cd206^+^ cells, as well as histology of lung and liver tissues in SAP mice after DNase I treatment (n=4 mice per group). Scale bar=50μm. **C)** Amylase levels and **D)** dsDNA levels in serum of SAP mice after DNase I treatment (n=4 mice per group). **E)** Immunofluorescence staining of DAPI (blue), Cd68 (red), and Mpo (green) and **F)** quantitative assessment of METs in the pancreata of SAP mice after DNase I treatment (n=4 mice per group). Scale bar=50μm. **G)** Western blot analysis of Gsdmd, N-Gsdmd, Mpo, and H3cit in the pancreata of SAP mice after DNase I treatment (n=4 mice per group). Statistical significance for **B, C, D** and **F** was determined using unpaired Student's t-tests. Data are presented as mean ± SD. Statistical significance: *P < 0.05, **P < 0.01, ***P < 0.001, ns. not significant. SAP, severe acute pancreatitis; METs, macrophage extracellular traps.

To further explore the role of METs in the pathophysiology of SAP, we administered DNase I intraperitoneally following SAP induction (Figure 2A). Histologically, DNase I administration attenuated pancreatic, pulmonary, and hepatic tissue damage and reduced macrophage infiltration in the spleen (Figure 2B). Serum amylase levels were also significantly decreased (Figure 2C). We then assessed the efficacy of METs clearance of DNase I administration. Following DNase I treatment, we observed a significant reduction in serum dsDNA levels, as well as a marked decrease in the number of METs in pancreatic tissues, identified by co-labeling with Cd68 and Mpo (Figure 2D-F). Western blot analysis further confirmed the significant downregulation of Mpo and H3cit expression following DNase I treatment (Figure 2G). Together, these results indicate that METs play a pivotal role in the progression of SAP and the associated systemic inflammatory response.

### The ROS-GSDMD Signaling Regulates the Formation of METs during SAP

**Figure 3:**
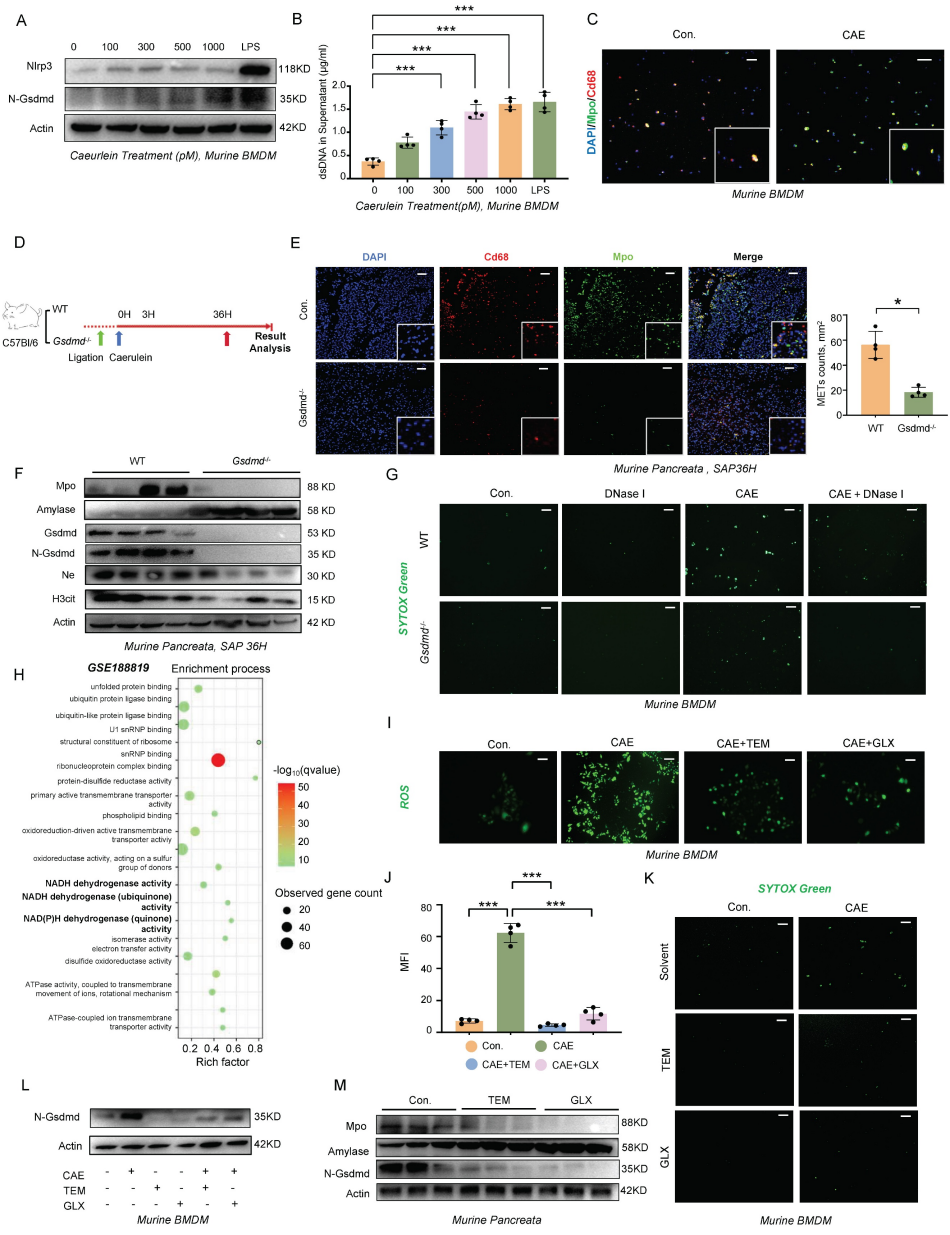
** The ROS-GSDMD signaling regulates the formation of METs in mice during SAP**. **A)** Western blot analysis of BMDMs and **B)** dsDNA levels in the supernatant after stimulation with a concentration gradient of caerulein and LPS for 4 hours (Performed in triplicate). **C)** Immunofluorescence staining of DAPI (blue), Cd68 (red), and Mpo (green) of BMDMs after stimulation with caerulein (500 pM) for 4 hours. Scale bar=25μm. **D)** *Gsdmd*^-/-^ mice were generated for SAP modeling. **E)** Immunofluorescence staining of DAPI (blue), Cd68 (red), and Mpo (green) in the pancreata of WT and *Gsdmd*^-/-^ mice at 36 hours after SAP induction, with MET counts also shown (n=4 mice per group). Scale bar=50μm. **F)** Western blot analysis of Mpo, Amylase, Gsdmd, N-Gsdmd, NE, and H3cit in the pancreata of WT and *Gsdmd*^-/-^ mice at 36 hours after SAP induction (n=4 mice per group).** G)** SYTOX Green staining of BMDMs from wild-type mice and *Gsdmd*^-/-^ mice treated with caerulein (500 pM) and/or DNase I (20 nM). Scale bar=50μm. **H)** GO enrichment analysis of differentially expressed genes in macrophages from pancreatitis mice compared to controls. **I)** Representative pictures and **J)** Quantitative analysis of ROS in BMDMs treated with caerulein (500 pM) for 4 hours, with and without Tempol (20 nM) and GLX481304 (20 nM) co-treatment. Scale bar=50μm. **K)** SYTOX Green staining of BMDMs from wild-type mice treated with caerulein (500 pM) with and without Tempol (20 nM) and GLX481304 (20 nM) co-treatment. Scale bar=50μm. **L)** Western blot analysis of N-Gsdmd in BMDMs from wild-type mice treated with caerulein (500 pM) with and without Tempol (20 nM) and GLX481304 (20 nM) co-treatment. **M)** Western blot analysis of Mpo, Amylase, and N-Gsdmd in the pancreata of SAP mice with and without Tempol and GLX481304 treatment. Statistical significance for **B** and **J** was analyzed using a one-way ANOVA with multiple comparisons test, and **E** was assessed using unpaired Student's t-tests. Data are presented as mean ± SD. Statistical significance: *P < 0.05, ***P < 0.001. CAE, caerulein treatment; LPS, Lipopolysaccharide treatment; TEM, Tempol treatment; GLX, GLX481304 treatment; SAP, severe acute pancreatitis; BMDMs, bone marrow-derived macrophages.

The relationship between pyroptosis and METs formation has not been previously reported. To investigate this potential association, we isolated bone marrow-derived macrophages (BMDMs) from wild-type mice and stimulated them with varying concentrations of caerulein for 4 hours. Our results revealed a dose-dependent increase in the cleaved form of Gsdmd following treatment (Figure 3A), accompanied by a corresponding increase in METs formation and dsDNA levels in the cell supernatant (Figure 3B, C). BMDMs treated with lipopolysaccharide (LPS) served as a positive control. We then induced SAP in both *Gsdmd*^-/-^ and wild-type mice using the same method (Figure 3D). Histological analysis of the pancreas and serum amylase levels showed an attenuated phenotype in *Gsdmd*^-/-^ mice compared to wild-type controls (Figure S1A, B). A significant decrease was observed in the number of Cd68 and Mpo co-labeled macrophages in pancreatic tissue and in serum dsDNA levels in *Gsdmd*^-/-^ mice (Figure 3E; Figure S1C). Additionally, protein expression levels of Mpo, H3cit, and Elastase were reduced, while amylase expression was restored in *Gsdmd*^-/-^ mice (Figure 3F). We further isolated BMDMs from both *Gsdmd*^-/-^ and wild-type mice and demonstrated that caerulein failed to induce METs formation, as assessed by SYTOX Green staining, in BMDMs derived from *Gsdmd*^-/-^ mice (Figure 3G). Collectively, these findings indicate that METs formation in the mouse pancreas during SAP is dependent on Gsdmd.

Several studies have highlighted the link between reactive oxygen species (ROS) production and the formation of ETs 9-11. Single-cell transcriptomic profiling (GSE188819) revealed significant enrichment of the NADH dehydrogenase activity pathway (FDR<0.01) in pancreatitis-associated macrophages, mechanistically linking mitochondrial ROS generation to disease progression (Figure 3H). Substantiating this finding, caerulein stimulation in WT BMDMs triggered ROS accumulation in a dose-responsive manner (Figure 3I,J). Pharmacological ROS inhibition using Tempol (a superoxide dismutase mimetic) and GLX481304 (a selective NOX inhibitor) concurrently attenuated METs formation (Figure 3K) and suppressed GSDMD-N terminal cleavage (Figure 3L). In addition to exogenous caerulein stimulation, we investigated how Damage-Associated Molecular Patterns (DAMPs) released from dying acinar cells influence METs formation by establishing an in vitro pancreatitis model using conditioned medium from injured acinar cells. Following caerulein stimulation and thorough washing to remove residual peptide, we collected injury-conditioned supernatants from cultured acinar cells (Figure S2A). Fluorescence microscopy and ROS probe analysis revealed that treatment of BMDMs with this conditioned medium significantly increased intracellular ROS levels (Figure S2B), demonstrating that damaged acinar cells potently induce ROS production in macrophages. This effect was attenuated by ROS scavengers Tempol and GLX481304, which concurrently reduced N-terminal GSDMD cleavage and decreased METs formation (Figure S2B-D) -results that mirrored those observed in GSDMD-knockout BMDMs. Importantly, genetic ablation of GSDMD did not affect ROS accumulation under either caerulein or conditioned medium stimulation (Figure S2E, F), providing conclusive evidence that ROS acts upstream of pyroptosis signaling in this pathway. Then, we further validated the therapeutic potential of ROS modulation *in vivo* using a murine SAP model. Pharmacological intervention with ROS-targeting compounds produced significant protective effects across multiple organ systems, as evidenced by reduced serum amylase levels (Figure S3A), ameliorated pancreatic histopathological damage, and improved injury scores in both liver and lung tissues (Figure S3B). At the molecular level, treatment effectively suppressed pancreatic N-terminal GSDMD cleavage (Figure 3M) and decreased circulating METs markers (Figure S3C, D). These findings conclusively demonstrate that ROS serve as crucial upstream regulators of GSDMD-mediated METs formation in the pathogenesis of SAP.

### METs Regulate CARS during SAP Progression

**Figure 4:**
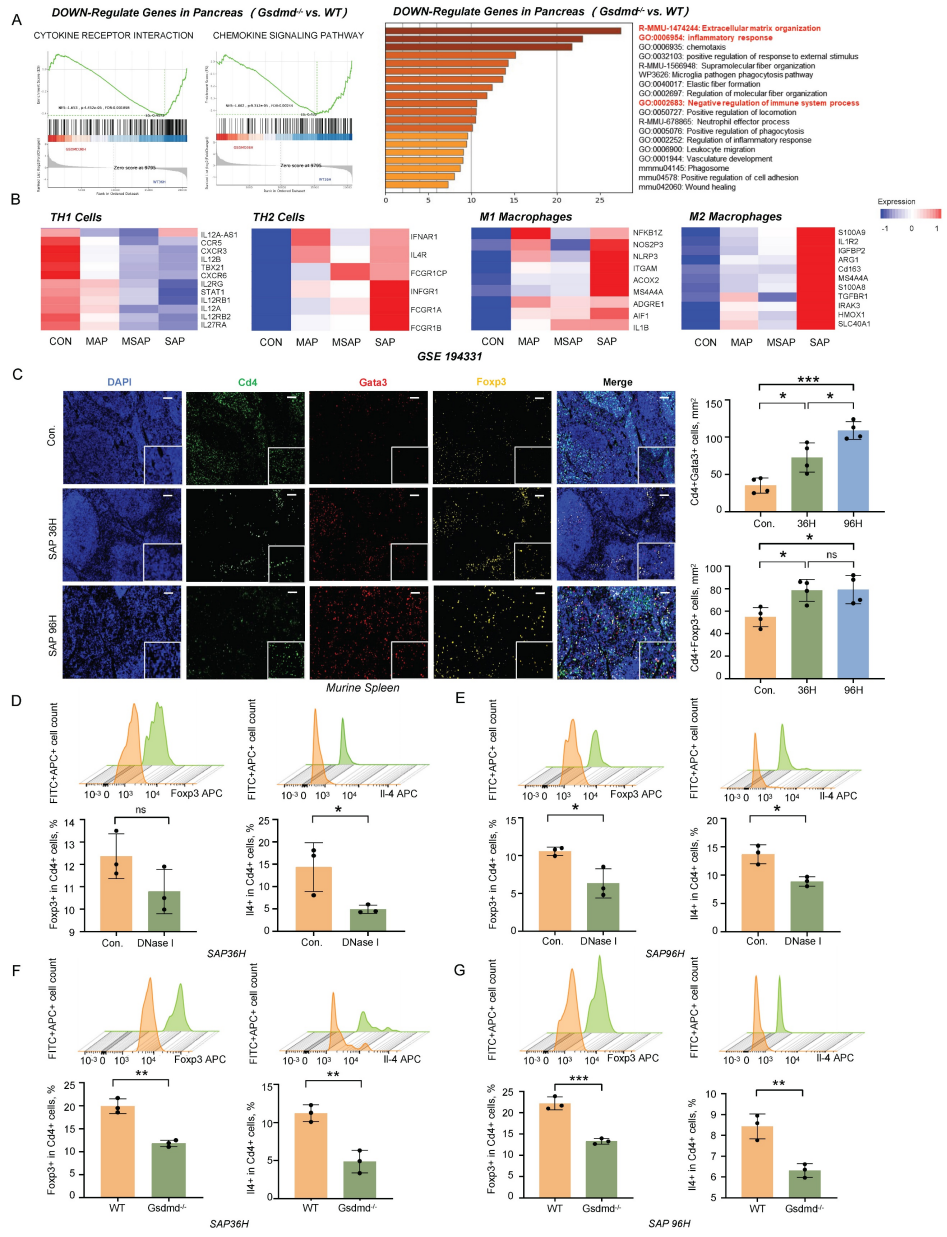
**METs regulate CARS during SAP progression. A)** GSEA analysis (left panel) and GO/KEGG-enrichment analysis (right panel) of transcriptomic data from pancreatic tissues of wild-type and *Gsdmd*^-/-^ mice after SAP induction. **B)** Markers related to Th1 cells, Th2 cells, M1 macrophages, and M2 macrophages in the peripheral blood of healthy controls and patients with different severities of acute pancreatitis (MAP, MSAP, and SAP) were examined using previously published RNA-seq data (GSE194331). **C)** Immunofluorescence staining of DAPI (blue), Cd4 (green), Gata3 (red), and Foxp3 (gold) in the spleen of control and SAP mice (n=4 mice per group). Scale bar=50μm. Quantitative analysis of Cd4^+^Gata3^+^ cells and Cd4^+^Foxp3^+^ cells is also included. **D, E)** Flow cytometric analysis of Treg cells and Th2 cells in the spleen of SAP mice, with and without DNase I treatment, at 36 hours and 96 hours post-modeling (n=3 mice per group). **F, G)** Flow cytometric analysis of Treg cells and Th2 cells in the spleen of wild-type and *Gsdmd*^-/-^ mice at 36 hours and 96 hours after SAP induction. (n=3 mice per group) Statistical significance for **C** was analyzed using a one-way ANOVA with multiple comparisons test, and **D-G** were calculated using unpaired Student's t-tests. Data are presented as mean ± SD. Statistical significance: *P < 0.05, **P < 0.01, ***P < 0.001, ns. not significant. CARS, Compensatory Anti-Inflammatory Response Syndrome; METs, macrophage extracellular traps; MAP, mild acute pancreatitis; MSAP, moderate to severe acute pancreatitis; SAP, severe acute pancreatitis.

During SAP, the systemic inflammatory response shifts from a state of SIRS to CARS 12. Using bulk RNA sequencing, we observed upregulation of inflammatory response pathways and immune-suppressive pathways in the pancreatic tissues of wild-type mice compared to *Gsdmd*^-/-^ mice during SAP (Figure 4A). Additionally, we analyzed a previously reported RNA sequencing dataset from the peripheral blood of pancreatitis patients (GSE194331). Our analysis revealed that the expression of markers for immune-suppressive Th2 cells and M2 macrophages increased in accordance with the severity of pancreatitis (Figure 4B). Immunofluorescent staining of Cd4, Gata3, and Foxp3 in the spleen following SAP induction showed an increasing number of regulatory T cells (Tregs) and Th2 cells as SAP progressed (Figure 4C). These findings suggest that an anti-inflammatory response mediated by the adaptive immune system is activated during the progression of SAP.

To investigate the impact of METs on the immune response during SAP, we performed flow cytometry analysis on splenic cells following DNase I treatment and *Gsdmd* knockout in mice with SAP. At 36h post-SAP modeling, the DNase I-treated group exhibited a downward trend in Treg differentiation, though this change was not statistically significant (Figure 4D). Meanwhile, Th2 cell differentiation was significantly decreased in this group (Figure 4D). By 96h, the decline in Treg cells become significant, and the reduction in Th2 cells persisted (Figure 4E). In *Gsdmd*^-/-^ mice, Treg cell differentiation was markedly lower than that in wild-type mice at 36h post-SAP induction (Figure 4F). This difference was sustained at 96h, with *Gsdmd*^-/-^ mice still showing significantly reduced Treg cell differentiation compared to WT controls (Figure 4G). Additionally, Th2 cell differentiation was also significantly diminished in *Gsdmd*^-/-^ mice at both time points (Figure 4F, G). Collectively, these findings indicate that METs and pyroptosis signaling disturb the balance between SIRS and CARS during murine SAP. The observed trends in the DNase I-treated group and the more pronounced changes in *Gsdmd*^-/-^ mice suggest that clearing METs or blocking pyroptosis may mitigate the CARS response, as evidenced by the altered differentiation of Treg and Th2 cells.

### METs Stimulate the Release of IL-33 from Acinar Cells via the cGAS-STING-GSDMD pathway

**Figure 5:**
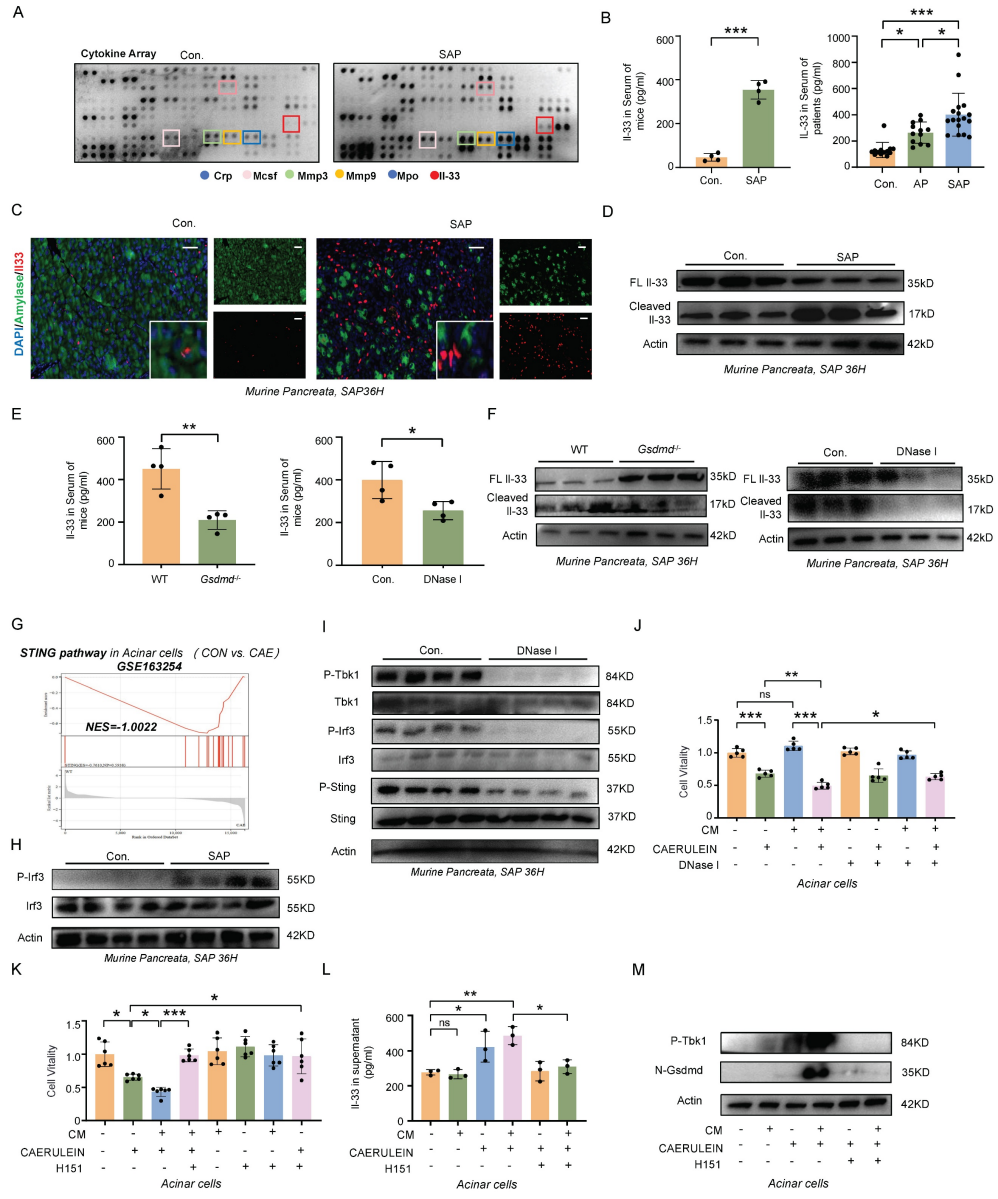
** METs stimulate the release of IL-33 from acinar Cells via the cGAS-STING-GSDMD pathway. A)** Cytokine array analysis of serum from control and SAP mice. **B)** ELISA analysis of Il-33 in serum of control and SAP mice (left panel, n=4 mice per group), and IL-33 in serum from healthy controls and patients with AP and SAP (right panel, n=12~17 subjects per group). **C)** Immunofluorescence staining of Amylase (green) and Il-33 (red) in the pancreata of control and SAP mice (n=4 mice per group). Scale bar=25μm. **D)** Western blot analysis of full-length Il-33 and cleaved Il-33 in the pancreata of control and SAP mice (n=3 mice per group).** E)** ELISA analysis of Il-33 levels in the serum of wild-type and *Gsdmd*^-/-^ mice following SAP induction, as well as ELISA analysis of Il-33 levels in the serum of SAP mice, comparing those with and without DNase I treatment. **F)** Western blot analysis of full-length Il-33 and cleaved Il-33 in the pancreata of wild-type and *Gsdmd*^-/-^ mice after SAP induction (n=3 mice per group) and in the pancreata of SAP mice with and without DNase I treatment (n=3 mice per group). **G)** GSEA enrichment analysis of the STING pathway in injured acinar cells, with data from GSE163254. **H)** Western blot analysis of Irf3 and phosphorylated Irf3 in the pancreata of control and SAP mice (n=4 mice per group). **I)** Western blot analysis of the total and phosphorylated forms of Tbk1, Irf3, and Sting in the pancreata of SAP mice with and without DNase I treatment (n=4 mice per group). **J-K)** CCK-8 viability assays of acinar cells after conditioned medium (CM) with BMDMs and caerulein treatment, with or without DNase I and H151 co-treatment. **L)** ELISA analysis of Il-33 in supernatant acinar cells after conditioned medium with BMDMs and caerulein treatment, with or without H151 co-treatment. **M)** Western blot analysis of phosphorylated Tbk1 and N-Gsdmd in acinar cells after conditioned medium with BMDMs and caerulein treatment, with or without H151 co-treatment. P values in left panel of **B** and** E** was calculated using unpaired Student's t-tests and P values in right panel of **B, J, K** and **L** was calculated using a one-way ANOVA with multiple comparisons test. Data are presented as mean ± SD. Statistical significance: *P < 0.05, **P < 0.01, ***P < 0.001, ns. not significant. CM, conditioned medium; H151, H151 treatment.

Circulating cytokines are critical in regulating the dynamic balance between SIRS and CARS. To explore the underlying mechanisms by which METs modulate CARS, we employed a Cytokine Array to examine changes in cytokine profiles in the serum of mice wih SAP. As shown in Figure 5A, levels of Mpo, a marker of METs, and interleukin-33 (Il-33), which mediates type 2 immune responses, were elevated in the serum of SAP mice. ELISA further confirmed that IL-33 was significantly increased in the serum of both SAP mice and patients (Figure 5B). In the pancreas of both human and mice, IL-33 primarily originates from acinar cells and is stored as a full-length protein within the nucleus under homeostatic conditions (Figure 5C, Figure S4A). Upon tissue injury, Il-33 is released (Figure 5C) and cleaved into its active form, which then binds to its specific receptor ST2 to regulate type 2 immunity. Here, we observed a significant increase in the cleaved form of Il-33 in murine pancreatic tissues following SAP induction (Figure 5D). However, in SAP mice treated with DNase I, there was a notable decrease in both the serum Il-33 levels and the cleaved form of Il-33 in tissues, accompanied by an increase in full-length Il-33 expression (Figure 5E, F). A similar trend was also observed in *Gsdmd*^-/-^ mice (Figure 5E, F). Based on these observations, we hypothesize that METs and pyroptosis may regulate the CARS state in SAP by modulating the release and cleavage of IL-33.

To analyze this process in more detail, we analyzed the transcriptome data of acinar cells following caerulein treatment (GSE163254) and identified significant enrichment of the cGAS-STING pathway (Figure 5G). Western blot analysis further confirmed an increase in phosphorylated Irf3, a downstream effector of the cGAS-STING pathway, in the tissues of SAP mice (Figure 5H). STING, an intracellular receptor, is capable of sensing signals triggered by exogenous DNA. Given that both the dsDNA within MET structures and the fragmented DNA released from damaged acinar cells can potentially activate the cGAS-STING pathway, we investigated the effects of DNase I treatment on this pathway. We found that DNase I treatment significantly reduced the phosphorylation levels of Tbk1, Irf3, and Sting in murine pancreatic tissues following SAP induction (Figure 5I). *In vitro*, DNase I treatment suppressed acinar cell damage mediated by both conditioned medium of macrophages and caerulein (Figure 5J). The cGAS-STING pathway is a critical signaling cascade that triggers pyroptosis, leading to the formation of pores in the acinar cell membrane through which IL-33 can be released. In acinar cells, treatment with H151, a STING signaling inhibitor, attenuated the conditioned medium and caerulein-induced increase in Gsdmd-mediated pyroptosis, Il-33 releases and cell damage (Figure 5K-M). Animal experiments further confirmed that blocking the STING signaling pathway alleviated the severity of SAP (Figure S5A-D). Collectively, these results demonstrate that METs regulate the STING pathway in acinar cells, leading to Gsdmd-mediated pyroptosis and subsequent release of IL-33 into the extracellular space.

### METs Cleave IL-33 into Highly Bioactive Isoforms to Induce Type-2 Immune Response

**Figure 6:**
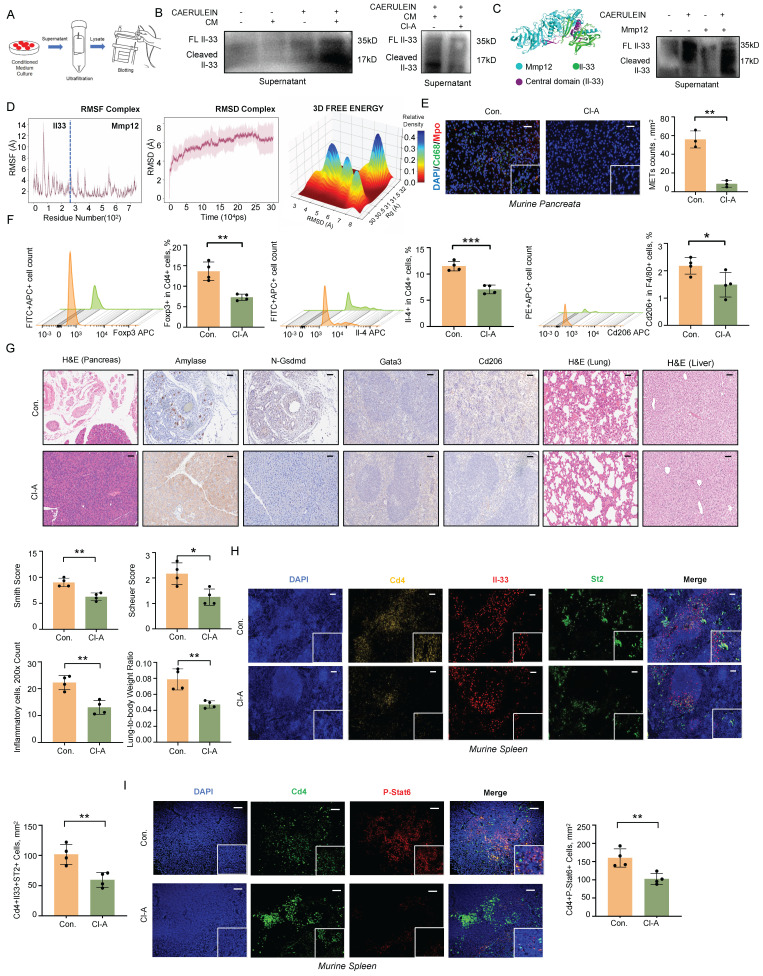
** METs cleave IL-33 into highly bioactive isoforms to induce type-2 immune response. A)** Workflow for obtaining supernatants from the co-culture system of acinar cells and BMDMs for western blot analysis. **B)** Western blot analysis of full-length and cleaved Il-33 in the cell supernatants of acinar cells after treatment with conditioned medium (CM) from BMDMs with and without caerulein (left panel) and Cl-Amidine (right panel). **C)** Molecular docking of Il-33 (green) and Mmp12 (blue) with the central domain of Il-33 highlighted in purple (left panel); Western blot analysis confirms the presence of cleaved Il-33 at approximately 17 kDa in the supernatant of acinar cells treated with both caerulein and Mmp12 (right panel). **D)** Molecular dynamics simulation of Mmp12 binding to Il-33, including: RMSF plot of the Il-33-Mmp12 complex depicting the fluctuations of individual atoms around their average positions in the complex over the simulation duration; RMSD plot of the Il-33-Mmp12 complex showing the deviation of atomic positions from the initial structure across 300 ns, reflecting the overall structural stability; 3D free energy landscape of the Il-33-Mmp12 complex, indicating regions of stability and conformational changes. **E)** Immunofluorescence staining of DAPI (blue), Cd68 (green), and Mpo (red) in the pancreata of SAP mice with and without Cl-Amidine treatment (n=3 mice per group). Scale bar=50μm. **F)** Flow cytometric analysis of Th2 cells, Tregs, and M2 macrophages in the spleen of SAP mice with and without Cl-Amidine treatment (n=4 mice per group). **G)** Representative images display pancreatic inflammation cell infiltration, Amylase and N-Gsdmd staining of pancreata, splenic immunostaining for Gata3^+^ and Cd206^+^ cells, as well as histology of lung and liver tissues in SAP mice after Cl-Amidine treatment (n=4 mice per group). Scale bar=50μm. **H)** Immunofluorescence staining of DAPI (blue), Cd4 (gold), Il-33 (red), and St2 (green) in the spleen of SAP mice with and without Cl-Amidine treatment (n=4 mice per group). **I)** Immunofluorescence staining of DAPI (blue), Cd4 (green), and p-Stat6 (red) in the spleen of SAP mice with and without Cl-Amidine treatment (n=4 mice per group). Statistical significance for **E, F, G, H** and **I** was determined using unpaired Student's t-tests. Data are presented as mean ± SD. Statistical significance: *P < 0.05, **P < 0.01, ***P < 0.001. CM, conditioned medium; Cl-A, Cl-Amidine treatment.

IL-33 is composed of three primary domains: an N-terminal domain for nuclear localization, a central domain that senses protease activity, and a C-terminal domain that mediates cytokine function. The protease-sensing domain can be targeted by inflammatory proteases, resulting in the cleavage of IL-33 into a short fragment with significantly enhanced activity 13. METs are web-like structures that abound with granulocytic proteases 14, 15, which may participate in the cleavage of IL-33. To test this hypothesis, we treated acinar cells with caerulein and conditioned medium from macrophages and used low-temperature ultrafiltration to obtain culture supernatants for western blot analysis (Figure 6A). Given that DNase I can degrade only dsDNA and has no effect on the protease components of METs, we employed the PAD inhibitor Cl-Amidine to specifically interfere with METs formation. Conditioned medium from macrophages pre-treated with Cl-Amidine significantly reduced the levels of cleaved Il-33 in the supernatant of acinar cells (Figure 6B). We further predicted the proteases within METs that potentially cleave Il-33.

Using the *ProsperousPlus* tool 16, we predicted potential IL33-degrading proteins among previously reported METs components 17, 18. The heatmap analysis revealed that Matrix Metalloproteinase 12 (Mmp12) exhibited superior cleavage potential in the central protease-sensing region of Il-33 compared to Mmp9, Elastase, and Cathepsin G (Figure S6A). Molecular docking studies indicated that Mmp12 (blue) has a potential interaction with Il-33 (green), with the central domain of Il-33 highlighted in purple (Figure 6C). Consistent with this prediction, treatment of acinar cells with recombinant Mmp12 in combination with caerulein significantly increased the levels of cleaved Il-33 in the cell supernatant (Figure 6C, Figure S6B). Molecular dynamics simulations further confirmed the interaction between Il-33 and Mmp12. Over a 300-nanosecond simulation, the Il-33-Mmp12 complex shifted from a highly dynamic to a more stable conformation, with notable structural alterations in the central domain of IL-33, suggesting potential enzymatic cleavage (Figure 6D, Figure S6C-E). Additionally, we observed an interaction between the Arg87 residue of Il-33 and a Glu residue near the active site of Mmp12 (Figure S6F). Previous research has shown that Glu residue plays a crucial role in enzymatic reactions, participating in nucleophilic attacks and peptide bond hydrolysis 19. *In vivo*, we also observed the colocalization of Mmp12 and Il-33 in pancreatic tissues from SAP mice (Figure S6G). Together, these findings indicate that MMP12 is a potential protease within METs capable of cleaving IL-33.

In SAP mouse models, Cl-Amidine treatment also confirmed the reduction of METs numbers (Figure 6E). Flow cytometry analysis revealed a significant decrease in anti-inflammatory responses associated Tregs, Th2 cells, and M2 macrophages following Cl-Amidine treatment (Figure 6F). Pathologically, both pancreatic injury and systemic inflammatory damage were improved after Cl-Amidine treatment (Figure 6G), accompanied by a reduction in the colocalization of Il-33 and St2 in spleen (Figure 6H). Moreover, phosphorylated Stat6, a downstream effector of St2, was also downregulated in Cd4^+^ T cells within spleen (Figure 6I). To conclude, these findings highlight the critical role of MET-derived MMP12 in the cleavage and activation of IL-33, thereby promoting systemic type 2 immune responses.

### IL-33/ST2 Mediates CARS and Exacerbates Multi-organ Damage during SAP

**Figure 7:**
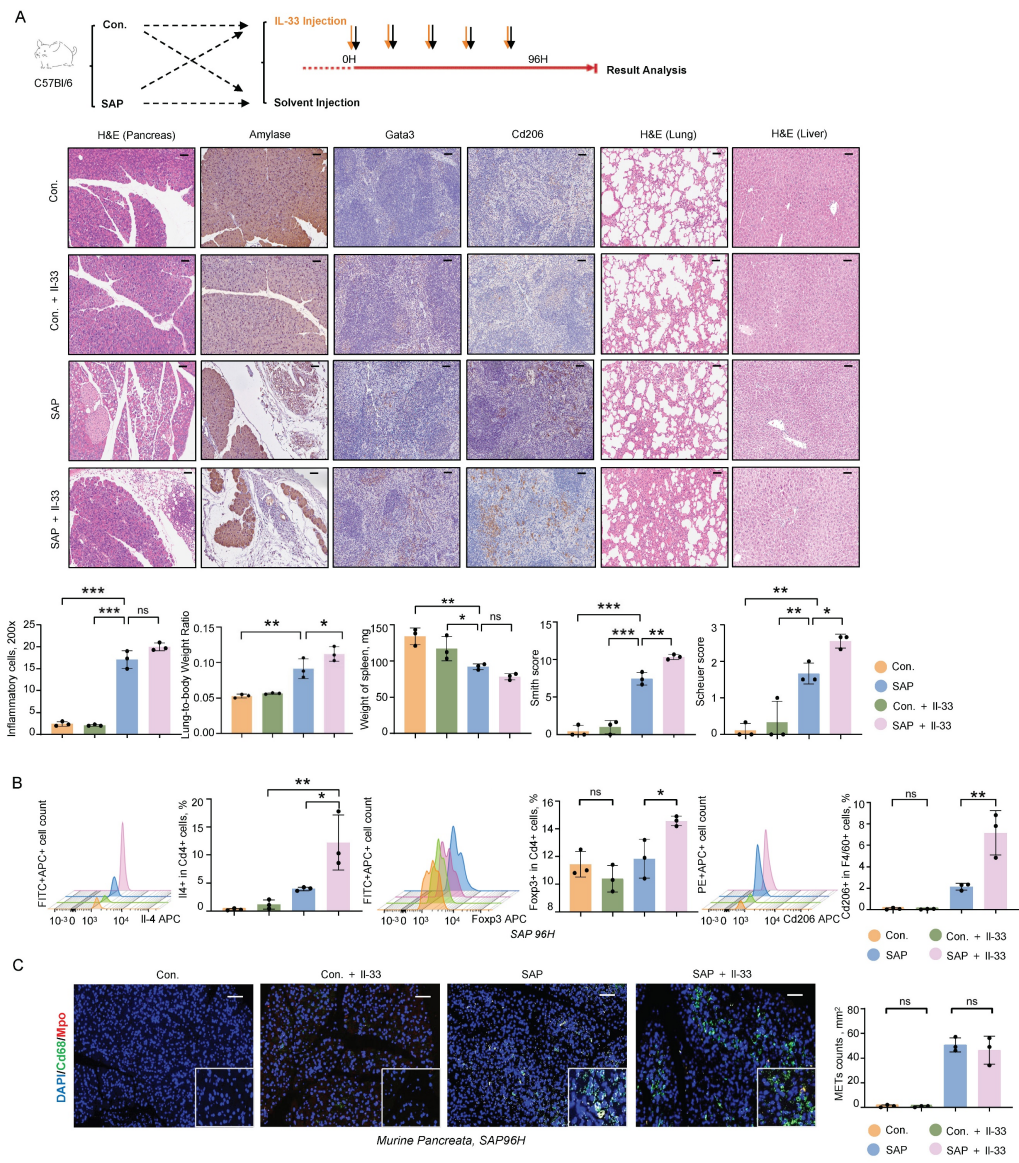
** IL-33/ST2 mediates CARS and exacerbates MODS during SAP. A)** Representative images of H&E, Amylase, and N-Gsdmd staining in the pancreas, Gata3 and Cd206 staining in the spleen, and H&E staining in the lungs and liver from control and SAP mice, with and without Il-33 treatment (upper panel), as well as statistical graphs depicting inflammatory cell counts in the pancreas, lung-to-body weight ratio, spleen weight, lung Smith score, and liver Scheuer score among these groups (lower panel, n=3 mice per group). Scale bar=50μm.** B)** Flow cytometric analysis of Th2 cells, Tregs, and M2 macrophages in the spleen of control and SAP mice, with and without Il-33 treatment (n=3 mice per group). **C)** Immunofluorescence staining of DAPI (blue), Cd68 (green), and Mpo (red) in the pancreata of control and SAP mice, with and without Il-33 treatment (n=3 mice per group). Scale bar=50μm. Statistical significance for **A, B** and **C** was determined using a one-way ANOVA with multiple comparisons test. Data are presented as mean ± SD. Statistical significance: *P < 0.05, **P < 0.01, ***P < 0.001, ns. not significant.

To further investigate the role of IL-33 in the progression of pancreatitis, we administered recombinant Il-33 intraperitoneally in an SAP mouse model. Il-33 treatment did not significantly affect inflammatory cell infiltration in pancreatic tissues but exacerbated damage in distant organs, including the lungs and liver, as evidenced by increased lung-to-body weight ratios and elevated Smith and Scheuer scores (Figure 7A). Flow cytometry analysis of the spleen revealed significant increases in Th2 cells, Tregs, and M2 macrophages following Il-33 treatment (Figure 7B). Additionally, immunofluorescence staining of METs showed that Il-33 had no impact on the formation of METs in the pancreatic tissues (Figure 7C). These results indicate that IL-33 is a critical factor exacerbating multi-organ damage and immune suppression during SAP.

## Discussion

SAP is a potentially fatal condition marked by severe inflammation and pancreatic injury, often leading to complications such as organ failure and tissue necrosis 1. The development of SAP can trigger a proinflammatory state (SIRS) and subsequently induce an immunosuppressive state (CARS), contributing to the complex pathophysiology of the disease 20. The latest understanding of SIRS/CARS regulation involves the process by which macrophages mediate the parallel activation of both the innate and adaptive immune systems during the early stages of SAP 3. Here, we demonstrate that METs promote type 2 immune responses and regulate CARS transition by facilitating IL-33 release from acinar cells via cGAS-STING signaling and enhancing IL-33 cleavage through MMP12. Our findings identify potential targets for predicting SAP outcomes and enabling early interventions.

The development of pancreatitis, especially the modulation of the systemic inflammatory response, involves a complex interplay between the innate and adaptive immune systems. During acute pancreatitis, macrophages are the predominant immune cells found within the inflamed pancreas, exhibiting high plasticity and possessing dual functions of both promoting and suppressing inflammation 7. In inflammatory disorders, web-like chromatin structures known as ETs are emerging as the forefront of research, playing a pivotal role in the regulation of inflammation 6. Similar to neutrophils, macrophages are known to release ETs (METs), yet their specific roles in pathological processes of SAP remain less well-characterized 21. Our findings revealed that macrophage-derived ETs are predominant in SAP, and occur in parallel with the multi-organ damage during the progression of SAP. The clearance of METs using DNase I effectively mitigated the severity of pancreatitis and reduced damage to distant organs.

ETs are typically formed in response to infections as part of the host's antibacterial defense mechanism. However, in sterile inflammation, such as pancreatitis, ETs can also can be activated by a range of stimuli, including cytokines and damage-associated molecular patterns (DAMPs) 22. There is growing evidence that excessive accumulation of ETs leads to tissue damage and prolonged inflammatory responses in acute pancreatitis 22, 23. However, existing research has primarily focused on the direct damaging effects of ETs on acinar cells, with a notable absence of studies investigating whether ETs play a role in modulating the immune system in acute pancreatitis. Here, we found that METs regulate the activation of Th2 cells in SAP, suggesting a role for METs in modulating the SIRS/CARS balance.

Emerging evidence underscores the pivotal role of interleukins (ILs) in pancreatitis pathogenesis across the disease spectrum, encompassing both pro-inflammatory mediators (e.g., IL-1β, IL-6, and IL-8) and anti-inflammatory regulators (e.g., IL-10 and IL-37) 24-26. To explore the mechanisms by which METs regulate Th2 cells activation during SAP, we performed a cytokine array analysis using serum samples of mice. During the process of SAP, we observed an upregulation of the IL-33 levels in serums, a well- known cytokine to regulate Th2-mediated immune responses 27. In non-inflammatory pancreas, IL-33 is retained within the nuclei of acinar cells, whereas during pancreatitis, it is released extracellularly. Given its established role in type 2 immunity and its marked elevation in SAP, IL-33 represents a critical focal point for investigation. The dynamic transition of IL-33 from nuclear sequestration to extracellular release during pancreatic inflammation suggests a key mechanistic link between tissue damage and immune modulation. Our research revealed that this process is associated with the activation of the cGAS-STING pathway in acinar cells and the subsequent induction of pyroptosis. Serving as a DNA sensor, STING is capable of triggering pro-inflammatory responses by recognizing DAMPs (DNA released from dying acinar cells), serving as a crucial step in the progression of acute pancreatitis 28. In addition to damaged acinar cells, our work demonstrated that METs themselves can also mediate STING activation as a type of DAMPs. Notably, the release of IL-33 from acinar cells occurs in parallel with the exacerbation of inflammation mediated by STING activation and pyroptosis. This suggests a hypothesis that STING not only serves as a pro-inflammatory signal but also functions as an upstream event regulating immunosuppression during SAP. Furthermore, we confirmed that METs, through their protease components, directly cleave extracellular IL-33 to significantly enhance its activity, which then activates Th2 cells via the ST2 receptor. Notably, experimental models can simulate some pancreatic injuries in SAP but fail to recapitulate overall clinical manifestations, especially complex systemic pathophysiological processes such as intestine-pancreas interactions and secondary infections 29, 30. Understanding these discrepancies is critical for interpreting results, advancing translational research, and refining models.

Both pro-inflammatory and anti-inflammatory signals are active in acute pancreatitis, and the outcome of the disease depends on the balance between hyperinflammation and hypoinflammation 31. Therefore, developing a therapeutic strategy that balances SIRS/CARS may represent the optimal choice for the treatment of SAP. Until now, the regulatory mechanisms of SIRS and CARS during SAP remain incompletely understood. Our work revealed that macrophage-derived web-like chromatin structures (METs) play a significant role in both pro-inflammatory and anti-inflammatory processes. Inhibiting the formation or facilitating the clearance of METs represents a promising interventional strategy to mitigate the progression and severity of SAP.

## Conclusion

In summary, this study provides new insights into the roles of METs in regulating immune responses during SAP. Briefly, METs are generated during the SAP process and interact with acinar cells as DAMPs to trigger the cGAS-STING signaling pathway and induce pyroptosis, resulting in the release of IL-33. The protease components of METs further cleave and activate IL-33, thereby promoting TH2 cell-mediated immune responses. In conclusion, our work has deepened the understanding of the regulatory mechanisms underlying the balance between SIRS and CARS during SAP, providing novel insights into clinical interventions for SAP.

## Materials and Methods

### Patient Sample Collection

Serum samples were collected from patients (aged 18-80 years) diagnosed with AP or SAP hospitalized at Nanjing Drum Tower Hospital, following the Atlanta Classification criteria. Inclusion required written informed consent, ability to complete sample collection and examinations. The exclusion criteria included patients with comorbidities that could significantly impact serum biomarker levels (e.g., decompensated liver cirrhosis, hepatic failure, malignant tumors, end-stage renal disease, or autoimmune disorders), pregnant or lactating women, and individuals with cognitive impairments that would hinder study compliance. Control samples were obtained from healthy individuals undergoing routine check-ups, matched for age and exclusion criteria. All serum samples were stored in a -80°C freezer for subsequent analyses. This study was approved by the Ethics Committee of Nanjing Drum Tower Hospital (Approval No.2024-736-01).

### Animal Experiments

C57Bl/6 mice were purchased from GemPharmtech company (Nanjing, China), and *Gsdmd*^-/-^ mice were generated using CRISPR-Cas9 technology by Shanghai Model Organisms Center (Shanghai, China). All mice were maintained at a specific pathogen-free (SPF) mouse facility of Nanjing University. To generate a mouse model of SAP, a procedure combining partial pancreatic duct ligation with caerulein injection was employed. In brief, mice were anesthetized using isoflurane and underwent laparotomy to facilitate partial ligation of the pancreatic duct proximal to the duodenum. Two days post-surgery, mice received an intraperitoneal injection of caerulein at a dose of 50µg/kg. The time of caerulein injection was designated as time zero, and mice were euthanized at 3 hours, 36 hours, or 96 hours post-injection. Then, the serum samples and tissues were collected for further analysis. The control group underwent a sham surgery procedure and received solvent injections. For treatment, mice were administered with DNase I (1mg/kg, HY-108882, MedChemExpress, Shanghai, China), Tempol (50mg/kg, HY-100561 , MedChemExpress, Shanghai, China), GLX481304 (50mg/kg, HY-153977 , MedChemExpress, Shanghai, China), H-151(10mg/kg, HY-112693, MedChemExpress, Shanghai, China), Cl-Amidine (75 mg/kg, HY-100574A , MedChemExpress, Shanghai, China) or Il-33 (50μg/kg, HY-P7218, MedChemExpress, Shanghai, China) intraperitoneally every 12 hours following surgical ligation of the pancreatic duct until the end of the experiment. All animal experiments were approved by the Animal Ethics Committee of Nanjing Drum Tower Hospital (Approval No.2024AE01026).

### Isolation and Culture of BMDMs

Eight-week-old C57BL/6 mice were euthanized under sterile conditions to harvest their femurs and tibias. The bone marrow cavities were flushed using a 21-gauge needle attached to a syringe filled with phosphate-buffered saline (PBS) containing 2% fetal bovine serum (FBS). The resulting cell suspension was filtered through a 70μm cell strainer to eliminate debris and large cell clusters. Following centrifugation at 1500 rpm for 5 minutes, the supernatant was discarded, and the cells were resuspended in Dulbecco's Modified Eagle Medium (DMEM) supplemented with 10% FBS. The cells were then seeded at a density of 2 ×10^5^ cells per well in six-well plates, with each well containing 2 ml of DMEM supplemented with 10% FBS and 30 ng/ml of macrophage colony-stimulating factor (M-CSF, CB34, Novoprotein, Shanghai, China). The cells were cultured at 37°C with 5% CO₂ for 7 days, with media changes every 2-3 days to maintain optimal nutrition and environmental stability. On day 7, the medium was replaced with fresh medium in preparation for subsequent experimental treatments.

### Isolation and Culture of Pancreatic Acinar Cells

Eight-week-old C57BL/6 mice were euthanized, and sterile pancreata were rapidly harvested and finely minced with ophthalmic scissors. The minced tissue was incubated in a solution containing collagenase II at 37°C for 30 minutes. After digestion, the tissue was filtered through a 70μm filter to remove undigested fragments. The filtrate was collected and resuspended in Waymouth's medium containing 10% FBS, trypsin inhibitor, and penicillin-streptomycin. Cells were plated in culture dishes and incubated at 37°C with 5% CO₂ for 1 day before harvesting suspended cells for subsequent experiments.

### Conditioned medium of BMDMs

Bone marrow cells were plated in 6-well plates at a density of 2×10^5^ cells per well and subsequently differentiated into BMDMs according to the aforementioned protocol. Prior to cell stimulation, the culture medium was exchanged with fresh DMEM. To induce the formation of METs, BMDMs were stimulated with 500 pM caerulein for 2 hours, with PBS serving as the control treatment. Then, supernatants were collected by centrifugation and used as the conditioned medium (CM) for stimulating acinar cells.

### Conditioned Medium of Injured Pancreatic Acinar Cells

Murine pancreatic acinar cells are cultured in a flask and stimulated with 500 pM caerulein for 4 hours. Once the stimulation is complete, the existing medium in the flask is carefully aspirated and replaced with fresh medium. After incubating the cells with the fresh medium for a certain period, the cells are gently washed, and the resulting medium is collected in a centrifuge tube. This collected medium is the conditioned medium of injured pancreatic acinar cells.

### Cell Viability Assay

Cell viability of acinar cells was assessed using the CCK8 assay kit (CK04, Dojindo, Kumamoto, Japan) according to the manufacturer's instructions.

### Western Blotting

Cells or tissues were lysed using RIPA buffer, and proteins were quantified using the BCA method. Proteins were separated by electrophoresis and transferred to PVDF membranes. Membranes were incubated with primary antibodies overnight, followed by incubation with horseradish peroxidase-conjugated secondary antibodies. A list of the antibodies and their dilution ratios used can be found in the Supplementary Materials. Immunoreactive bands were visualized using the ECL detection reagents (E423-01/02, Vazyme, Nanjing, China).

### Immunofluorescence

Cells or tissue sections (2μm) were fixed in 4% paraformaldehyde. For antigen retrieval, sections were treated with citrate buffer (pH 6.0) or EDTA-NaOH buffer (pH 8.0 or 9.0) according to the specifications of the primary antibodies. Sections were permeabilized with 0.2% Triton X-100 and blocked with 2% BSA in PBS. They were then incubated overnight at 4°C with primary antibodies, followed by fluorophore-conjugated secondary antibodies for 2 hours at room temperature. Nuclei were counterstained with DAPI for 10 minutes, and sections were mounted with an anti-fade medium and imaged using a Leica Thunder System.

For multiplex immunostaining (three-color staining), sections were incubated overnight at 4°C with the first primary antibody, followed by HRP-conjugated secondary antibodies for 50 minutes at room temperature. TSA reagents for red (TSA 570), green (TSA 480), or gold (TSA 620) fluorescence were added and incubated for 10 minutes. After TSA reaction, sections were washed extensively with TBST. This process was repeated for the second and third primary antibodies, with antigen retrieval performed before each new primary antibody. Finally, sections were imaged using a Nikon Eclipse C1 with NIKON DS-U3 system.

### SYTOX Green Staining

SYTOX Green staining was used to detect extracellular DNA of METs. An appropriate amount of SYTOX Green dye (S7020, Thermo Fisher Scientific Inc., Waltham, the USA) was added to the cell culture dish to achieve a final concentration of 1μM. The dish was then in the dark at room temperature for 15 minutes. After incubation, cells were washed three times with PBS to eliminate any excess dye and observed using EVOS M7000 (Thermo Fisher Scientific Inc., Waltham, the USA).

### H&E Staining, Immunohistochemistry, and Histological Examination

Mouse tissues were fixed, dehydrated, embedded, and sectioned for pathological evaluation. Sections of pancreatic, lung, and liver tissues were stained with H&E following standard staining protocols. H&E staining of pancreatic tissues was employed to quantify inflammatory cell infiltration counts in fields magnified 200×. The Smith lung injury scoring was used to assess lung tissue injury according to the method reported in the literature 32. In brief, the Smith score was evaluated based on four parameters: alveolar and interstitial inflammation and pulmonary edema; alveolar and interstitial hemorrhage; atelectasis; and hyaline membrane formation. Each of these parameters was assigned a score ranging from 0 to 4, where 0 represented no injury or injury affecting less than 25% of the field, 1 denoted injury affecting 25% of the field, 2 signified injury affecting 50% of the field, 3 indicated injury affecting 75% of the field, and 4 implied injury affecting more than 75% of the field. Finally, the total score was calculated and analyzed (ranged from 0-16). The Scheuer score was used to assess the severity of inflammation in liver tissues as described previously 33. Considering the actual hepatic pathological changes in SAP mice, this scoring system was set to a 0-4 range in the present study, where a score of 0 indicates no obvious inflammation, scores 1 to 4 represent a gradual increase in inflammation severity, and a score of 4 denotes the most severe inflammation. Meanwhile, tissue sections were also used for immunohistochemical analysis, and the antibodies and dilutions used in this study were listed in the Supplementary Materials. Positive area or cell count was quantified using ImageJ software. At least five random fields of view were examined for each section. All pathological assessments were conducted by two experienced pathologists who discussed discrepancies until consensus was reached to ensure accurate and reliable scoring.

### Flow Cytometry

Splenocytes were collected following the previously described procedure 34. To detect intracellular cytokine markers, including Il-4 and Ifn-γ, an intracellular cytokine stimulation and blocking agent (E-CK-A019, Elabscience, Wuhan, China) was used. Briefly, the splenocyte suspension was first incubated with an Fc receptor blocker at room temperature for 15 minutes, then with live/dead cell dye at room temperature in the dark for 10 minutes. The pre-treated cells were washed twice with PBS, incubated with an intracellular cytokine stimulation blocking agent at 37°C for 5 hours, then fixed and permeabilized at room temperature for 20 minutes followed by a PBS wash. Finally, the cells were incubated with labeled antibodies at 4°C in the dark for 30 minutes, washed, resuspended in PBS, and analyzed using a BD flow cytometer (Canto II, Becton, Dickinson and Company, Franklin Lakes, USA).

Foxp3 was detected using a fixation/permeabilization kit (E-CK-A108, Elabscience, Wuhan, China). After blocking Fc receptors and staining with live/dead cell dye, the cells were incubated with fluorescent antibodies. Subsequently, 1 mL of 1× Fixation Working Solution was added, and the mixture was incubated at 4°C for 30 minutes. The cells were then centrifuged at 600×g for 5 minutes, and the supernatant was discarded. The cells were treated twice with 2 mL of 1×Permeabilization Working Solution, with each treatment followed by centrifugation at 600×g for 5 minutes and supernatant discard. Finally, the cells were resuspended in 100μL of 1×Permeabilization Working Solution for nuclear staining and subsequent analysis.

### IL-33, Amylase and dsDNA Detection in Serum

Human and mouse serum samples were collected and employed for the detection of IL-33 using Quantikine ELISA kits specifically designed for human (EK0929, Boster Biological Technology, Wuhan, China) and mouse (EK0930, Boster Biological Technology, Wuhan, China), following the manufacturer's specified instructions. Amylase in mouse serum was measured using an automated biochemical analyzer (AU5800, Beckmancoulter, Brea, the USA) in the Medical Laboratory Department of Nanjing Drum Tower Hospital, following the instrument operation manual. For dsDNA detection, the Equalbit dsDNA HS (High Sensitivity) Assay Kit (EQ111-01/02, Vazyme, Nanjing, China) was used according to the directions provided by the manufacturer.

### Cytokine Array

To evaluate the dynamic changes in serum cytokines during the SAP process, the Proteome Profiler Mouse XL Cytokine Array Kit (ARY028, R&D Systems, Minneapolis, the USA) was used in strict accordance with the manufacturer's procedure.

### Il-33 Detection in Supernatant

To enhance the sensitivity and accuracy of Il-33 detection in supernatants, ultrafiltration concentration was implemented. A 3 kDa ultrafiltration device was meticulously prepared, ensuring thorough cleaning and sterilization to prevent contamination. Cold-preserved culture supernatants (2 ml) were carefully loaded into the ultrafiltration tube without introducing bubbles to safeguard the integrity of the ultrafiltration membrane. The tube was then centrifuged at 5000 rpm for 120 minutes at a low temperature. During this process, small molecules such as water and salt ions permeated through the membrane, while Il-33 was retained in the concentrate. Centrifugation was continued until the supernatant volume was reduced from 2 ml to 200 µl. The concentrated supernatant was then carefully transferred to a suitable microcentrifuge tube. Following RIPA lysis, the concentrated samples were analyzed using Western blotting.

### ProsperousPlus

To identify potential enzymes involved in the cleavage of Il-33 active fragments, we employed *ProsperousPlus*—a one-stop platform for accurate protease-specific substrate cleavage prediction and model construction, available as a web server (http://prosperousplus.unimelb-biotools.cloud.edu.au/) and standalone package. The FASTA-formatted amino acid sequence of mouse Il-33 obtained from Uniprot was input into the web server. After checking the names of the proteases of interest listed below and clicking "Submit", the system processed the sequence through a series of machine learning algorithms and output the cleavage sites along with corresponding scores. A default score of 0.5 or higher indicates that the cleavage site is meaningful.

### Protein Docking

To predict the interaction details between mouse-derived Il-33 and Mmp12 proteins, protein docking was conducted using the Chai-1 tool (lab.chaidiscovery.com). The PDB-formatted structural files of mouse Il-33 and Mmp12 were retrieved from UniProt and imported into the Chai-1 platform. The optimal docking results were subsequently visualized using PyMOL (DeLano Scientific LLC, the USA), which provided a clear depiction of the molecular interactions occurring between Il-33 and Mmp12.

### Molecular Dynamics Simulation

Molecular dynamics simulations were conducted to investigate the interaction mechanism between Il-33 and Mmp12. Simulations were performed using Amber 24 software (AMBER Software Administrator, University of California, San Francisco, the USA) with the ff19SB force field and TIP3P water model, adding 0.15M Na⁺ and Cl⁻ counterions. After energy minimization, the system was heated from 0K to 310.15K (37°C) over 500ps in the NVT ensemble. Following equilibration, the simulation was switched to the NPT ensemble at 1 bar pressure and 310.15K for 300ns, with a time step of 2 fs. Frames were saved every 10ps, and hydrogen-related covalent bonds were constrained using the SHAKE algorithm. To ensure reliable sampling, three rounds of unbiased repeats were conducted. Analysis methods included root-mean-square deviation (RMSD) and root-mean-square fluctuation (RMSF) analysis, which quantify structural differences and atomic fluctuations, respectively.

### RNA Sequencing

Wild-type and *Gsdmd*^-/-^ mice were sacrificed 36 hours after SAP induction and pancreatic tissues were harvested and preserved in RNA later solution (AM7024, Thermo Fisher Scientific Inc., Waltham, the USA). After RNA extraction, RNA sequencing was performed at Shanghai Applied Protein Technology Co., Ltd. (APTBIO, Shanghai, China) using the Illumina NovaSeq platform with paired-end 150bp reads.

### Single Cell Data Analysis

Single-cell RNA sequencing (scRNA-seq) data were obtained from GSE188819 in the Gene Expression Omnibus (GEO) database. The Seurat package (Satija Lab, New York University, New York, the USA) was used to filter low-quality cells and low-expressing genes, and normalize the data. Principal component analysis (PCA) was performed to reduce dimensionality and t-distributed stochastic neighbor embedding (t-SNE) were applied for visualization and clustering the resulting data. Cell annotations were based on the annotations provided within the dataset. Differential gene expression analysis was conducted across different cell clusters or groups. Gene Ontology (GO) enrichment analysis was performed using the clusterProfiler package (Yu Lab, Southern Medical University, Guangzhou, China) to identify significantly enriched pathway. Additionally, CellChat analysis 35 was employed to visualize cell-cell communication patterns.

### Reanalysis of Public RNA Sequencing Data

All public data of RNA sequencing used in this study were obtained from the Gene Expression Omnibus (GEO) database, with accession codes GSE163254 and GSE194331. Genes and samples with more than 50% NA values were removed to eliminate potential interference from missing data. Differential expression analysis was performed using the Limma package 36. GO-Enrichment and GSEA was performed using the clusterProfiler package (Yu Lab, Southern Medical University, Guangzhou, China), while heatmaps were generated using Hiplot PRO software (provided by Shanghai Tengyun Biotechnology). All bioinformatics analyses were based on R Studio. We also used Metascape (https://metascape.org/gp/index.html#/main/step1) for Gene Ontology (GO) and Kyoto Encyclopedia of Genes and Genomes (KEGG) enrichment scoring.

### HPA Database Analysis

High-resolution images of IL-33 expression in human pancreas annotated with IHC pictures for human IL-33 were obtained from HPA database (proteinatlas.org).

### Statistical Analysis

Data are expressed as mean ± standard deviation (SD). Comparisons between two groups were performed using a two-tailed Student's t-tests. For comparisons involving three or more groups, one-way analysis of variance (ANOVA) was used, followed by post-hoc tests where appropriate. P values less than 0.05 were considered statistically significant. All statistical analyses were conducted using GraphPad Prism software (9.0, Boston, the USA) or RStudio (4.4.0).

## Supplementary Material

Supplementary figures and table.
