# Supplementary material for: Macrophage Extracellular Traps Modulate the Compensatory Anti-inflammatory Response Syndrome through IL-33/ST2 Signaling in Severe Acute Pancreatitis
Source: Int J Biol Sci. 2025 Sep 22;21(14):5989–6006. doi: 10.7150/ijbs.116740 (PMC12594551; doi:10.7150/ijbs.116740)
Supplement: Supplementary file 1 — Supplementary figures and table. [file ijbsv21p5989s1.pdf]

Supplementary Materials

I: Antibodies and dilution

Primary/Secondary antibody	Company	Cat. No	Dilution
NLRP3	CST	15101	1:1000
GSDMD	Abcam	ab219800	1:1000
N-GSDMD	Affinity	DF13758	1:1000 for WB, 1:500 for IHC
AMYLASE	CST	3796	1:1000 for WB, 1:500 for IHC
ACTIN	Proteintech	66009-1-Ig	1:1000
MPO	Abcam	ab208670	1:1000 for WB, 1:500 for IF
H3CIT	Abcam	ab281584	1:1000
NE	Abcam	ab131260	1:1000
Caspase4	Proteintech	11856-1-AP	1:1000
Cleaved-Caspase-1	Affinity	AF4005	1:1000
IL-33 (FOR WB)	Abclonal	A8096	1:1000
IL-33 (FOR IHC)	Abways	CY5105	1:100
ST2	Abcam	ab194113	1:500
P-STAT6	Affinity	AF3301	1:500
MMP12	Dreambio	YM-Y23544	1:1000
LY6G	Abclonal	A22270	1:100
GATA3	Affinity	AF6233	1:100
TBET	Affinity	DF7759	1:100
CD68	Abcam	ab303565	1:500
CD68	Abcam	ab53444	1:500
CD86	Abways	DF6332	1:500
CD206	Proteintech	18704-1-AP	1:500
TBK1	Abways	CY5145	1:1000
P-TBK1	Abways	CY8364	1:1000
STING	Proteintech	19851-1-AP	1:1000
P-STING	Affinity	AF7416	1:1000
IRF3	Abways	CY5779	1:1000
P-IRF3	Proteintech	29528-1-AP	1:1000
FITC Anti-Mouse CD4 Antibody[RM4-5]	Elabscience	E-AB-F1353C	1:20
APC Anti-Mouse Foxp3 Antibody[3G3]	Elabscience	E-AB-F1238E	1:20
APC Anti-Mouse IL-4 Antibody[11B11]	Elabscience	E-AB-F1204E	1:20
PE Anti-Mouse F4/80	Elabscience	E-AB-F0995D	1:20

Antibody[CI:A3-1]			
APC Anti-Mouse CD206/MMR Antibody[C068C2]	Elabscience	E-AB-F1135E	1:20
PE Anti-Mouse IFN-γ Antibody[XMG1.2]	Elabscience	E-AB-F1101D	1:20
Alexa Fluor 488-labeled Goat Anti-Rabbit IgG(H+L)	Beyotime	A0423	1:200
Alexa Fluor 488-labeled Goat Anti-Mouse IgG(H+L)	Beyotime	A0473	1:200
Cy3-labeled Goat Anti-Rat IgG(H+L)	Beyotime	A0507	1:200
Cy3-labeled Goat Anti-Rabbit IgG (H+L)	Beyotime	A0562	1:200
FITC-labeled Goat Anti-Mouse IgG (H+L)	Beyotime	A0568	1:200
Anti-rabbit IgG, HRP-linked Antibody	CST	7074	1:5000
Anti-mouse IgG, HRP-linked Antibody	CST	7076	1:5000
HRP-labeled Goat Anti-Rabbit IgG(H+L)	Beyotime	A0208	1:200
HRP-labeled Rabbit Anti-Mouse IgG(H+L)	Beyotime	A0354	1:200

II: Supplementary Figures and Figure Legend

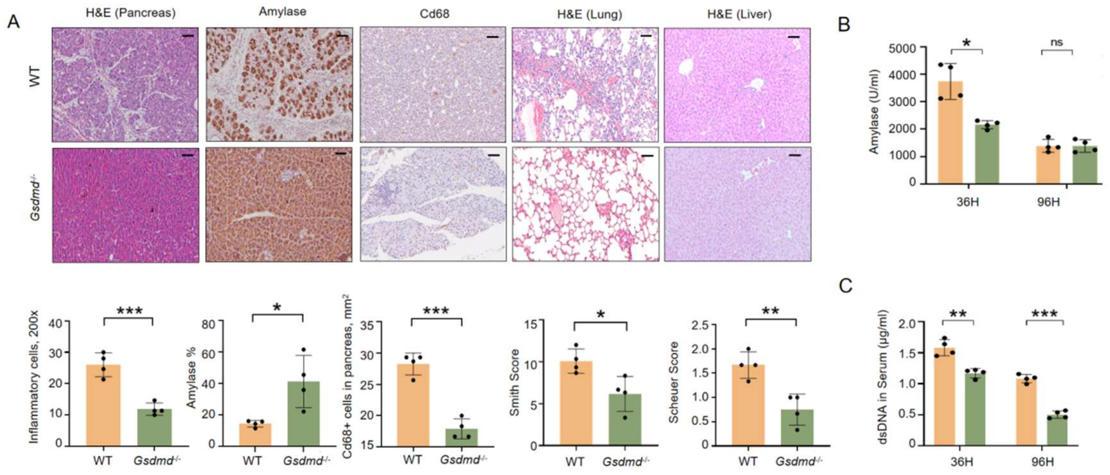

Figure S1. GSDMD Knockout Alleviates SAP in Mice A) Representative images and quantitative assessments show pancreatic inflammation cell infiltration, amylase staining of pancreata, pancreatic immunostaining for $Cd68^+$ cells, as well as histological scoring of lung and liver tissues in wild-type and *Gsdmd*$^{-/-}$ mice after SAP induction (n=4 mice per group).

Scale bar=50μm. **B)** Serum amylase measured in wild-type and *Gsdmd*$^{-/-}$ mice after SAP induction (n=4 mice per group). **C)** dsDNA levels measured in wild-type and *Gsdmd*$^{-/-}$ mice after SAP induction (n=4 mice per group). Statistical significance for **A, B** and **C** was determined using unpaired Student's t-tests. Data are presented as mean ± SD. Statistical significance: *P < 0.05, **P < 0.01, ***P < 0.001, ns. not significant.

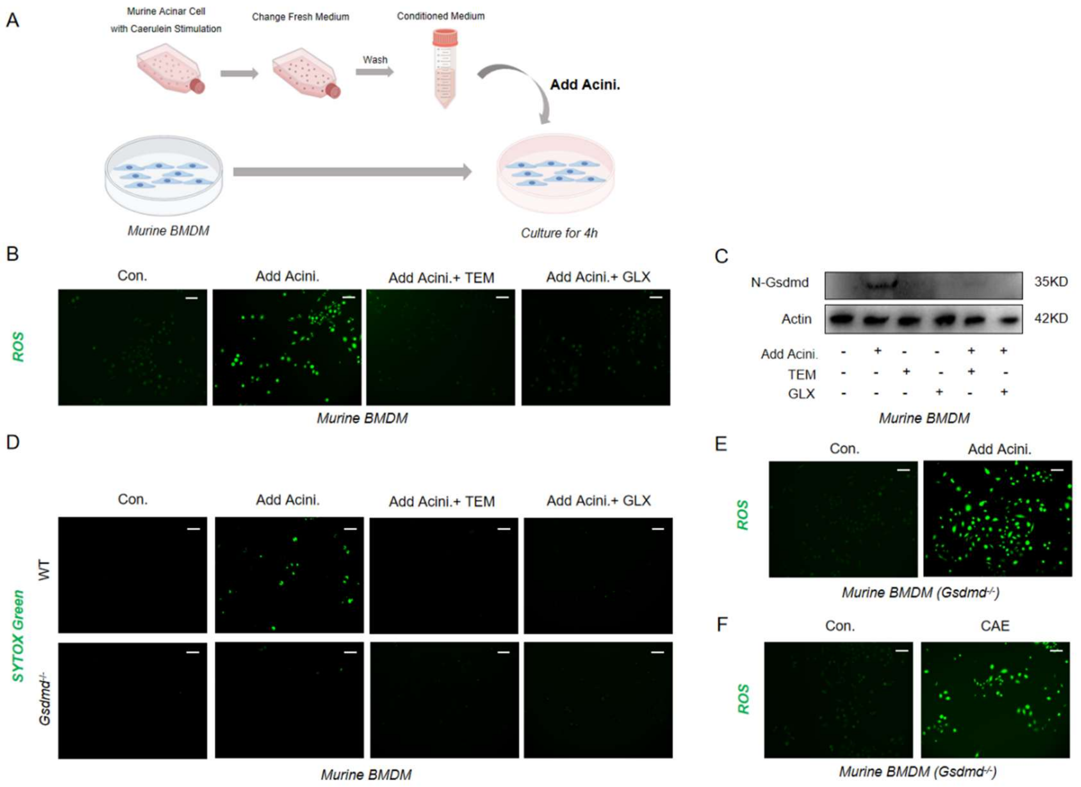

Figure S2. Injured pancreatic acinar cells mediate METs release via the ROS-GSDMD axis. A) Schematic of the procedure for obtaining conditioned medium from injured acinar cells. Created with Figdraw.com. **B)** Representative images of reactive oxygen species (ROS) in wild-type bone marrow-derived macrophages (BMDMs) treated with conditioned medium from injured acinar cells for 4 hours, with or without co-treatment with Tempol (20 nM) and GLX481304 (20 nM). Scale bar=50μm. **C)** Western blot analysis of N-terminal Gasdermin D (N-Gsdmd) in BMDMs treated with conditioned medium from injured acinar cells, Tempol (20 nM), GLX481304 (20 nM), or combinations of these factors. **D)** Representative images of SYTOX Green in wild-type or *Gsdmd* knockout BMDMs treated with conditioned medium from injured acinar cells for 4 hours, with or without co-treatment with Tempol (20 nM) and GLX481304 (20 nM). Scale bar=50μm. **E, F)** Representative images of ROS in *Gsdmd* knockout BMDMs treated with conditioned medium from injured acinar cells or caerulein (500 pM) for 4 hours. Scale bar=50μm.

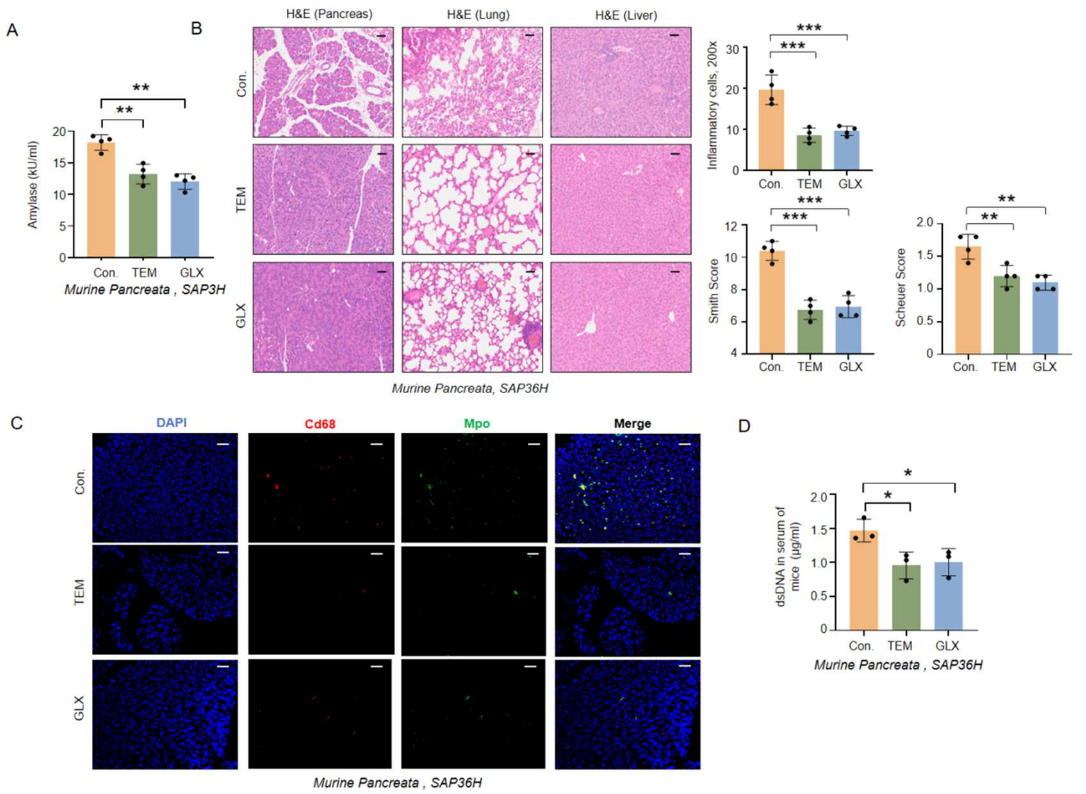

Figure S3. Pharmacological Inhibition of ROS Suppresses METs Formation and Alleviates Pancreatic Injury. A) Serum amylase levels in SAP mice treated with Tempol(TEM) or GLX481304(GLX) (n=4 mice per group). **B)** Representative images and quantitative analysis of H&E staining of mouse pancreas, lung and liver. (n=4 mice per group). **C)** Immunofluorescence images of DAPI (blue), Cd68 (red), and Mpo (green) in pancreata of SAP mice treated with Tempol or GLX481304 (n=4 mice per group). **D)** Serum dsDNA levels in SAP mice treated with Tempol or GLX481304 (n=3 mice per group). Statistical significance for **A, B** and **D** was determined using a one-way ANOVA with multiple comparisons test. Data are presented as mean ± SD. Statistical significance: *P < 0.05, **P < 0.01, ***P < 0.001. Scale bar=50μm.

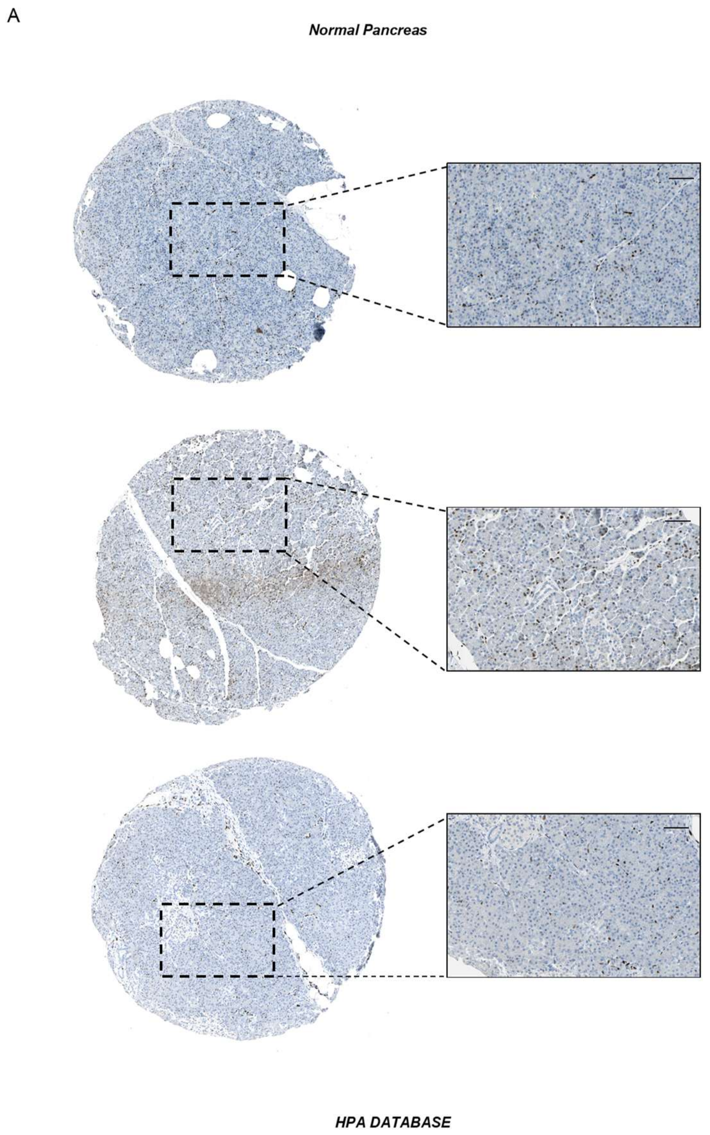

Figure S4. Expression of IL-33 in Human Normal Pancreas. A) Immunohistochemistry data from the Human Protein Atlas (HPA) database show that IL-33 is expressed in the nucleus of acinar cells in the normal human pancreas from three patients. Scale bar=50μm.

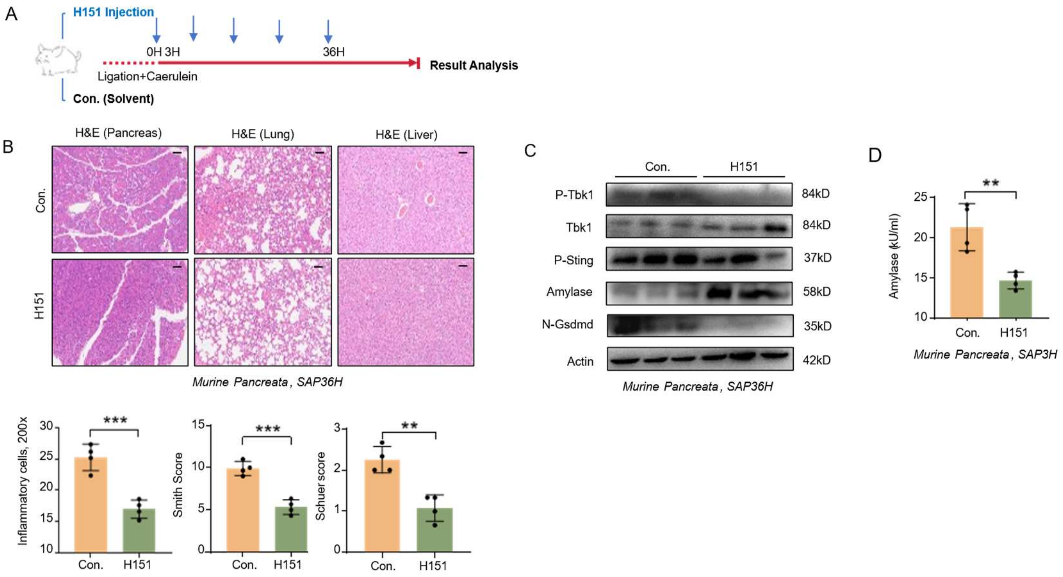

Figure S5. Pharmacological Inhibition of STING Signaling Pathway Alleviates Pancreatic Injury during SAP. A) Schematic diagram illustrating the establishment of the SAP mouse model and H151 administration procedures. **B)** Representative images and quantitative analysis of H&E staining of mouse tissues (n=4 mice per group). Scale bar=50μm. **C)** Western blot analysis of Amylase, N-Gsdmd, and phosphorylated forms of Tbk1 and Sting in the pancreata of SAP mice with and without H151 treatment (n=3 mice per group). **D)** Serum amylase levels in SAP mice with and without H151 treatment (n=4 mice per group). Statistical significance for **B** and **D** was determined using unpaired Student's t-tests. Data are presented as mean ± SD. Statistical significance: **P < 0.01, ***P < 0.001.

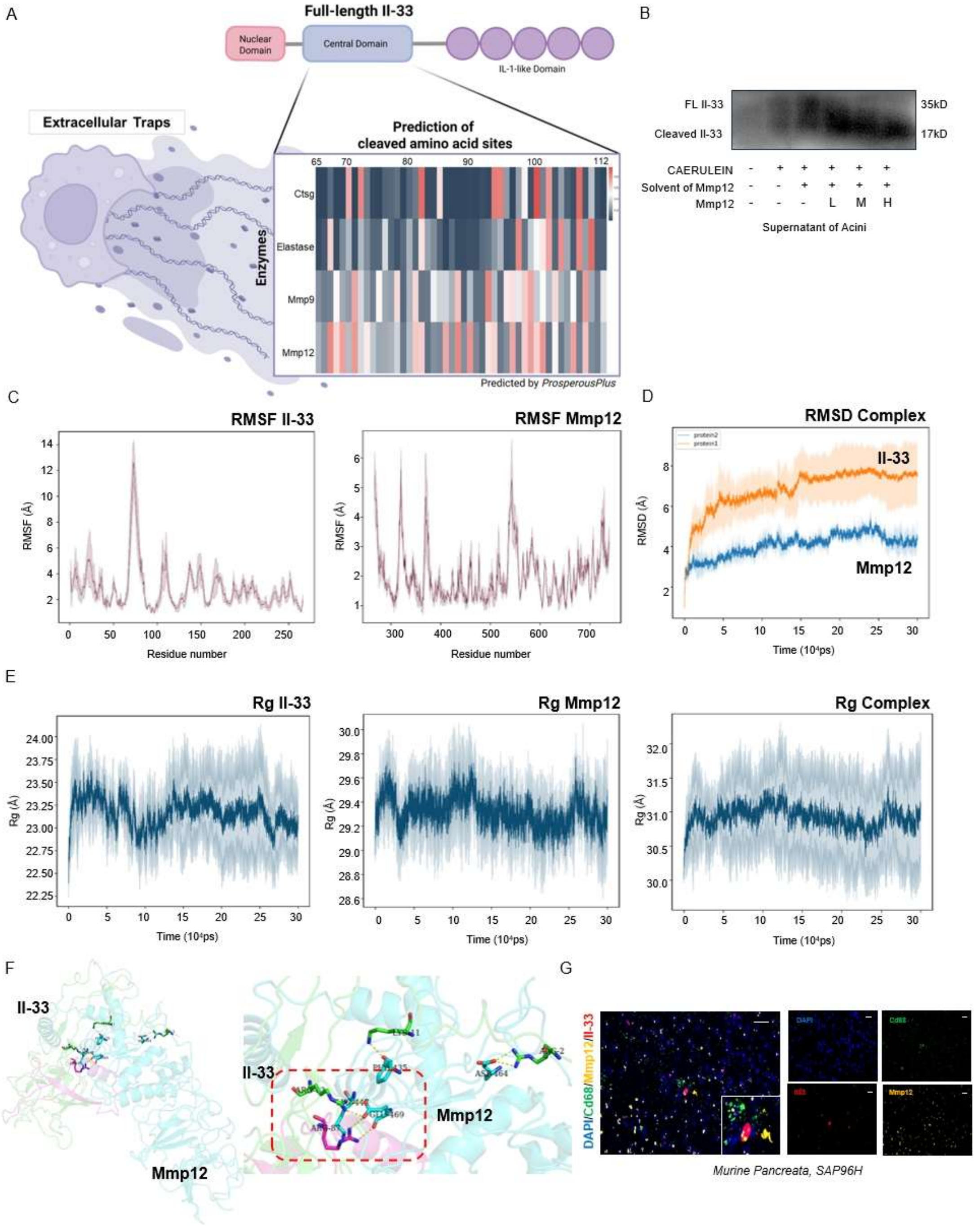

Figure S6. Prediction of the Cleavage of IL-33 by MMP12. A) The potential cleavage sites in the central domain of Il-33 were predicted using the *ProsperousPlus*. Created with Biorender.com. **B)** Western blot analysis demonstrating that addition of Mmp12 results in cleavage of Il-33 in injured acinar supernatants [L=low dose(10pM), M=medium dose(100pM), H=high dose(1nM)]. **C)** Root mean square fluctuation (RMSF) plots for individual residues of Il-33 (left) and Mmp12 (right) throughout the simulation. **D)** Root mean square deviation (RMSD) over time for the Il-33-Mmp12 complex, indicating stability during the simulation period. **E)** Radius of gyration (Rg) plots for Il-33, Mmp12, and their complex, illustrating changes in compactness over time. All molecular dynamics simulations

were performed in triplicate to ensure reliability. **F)** Molecular dynamics simulation snapshots showing the interaction between Il-33 (green) and Mmp12 (blue). The binding interface is highlighted by a red dashed box. **G)** Immunofluorescence staining of pancreatic tissues following SAP induction showing colocalization of Cd68 (green), Mmp12 (gold), and Il-33 (red). Nuclei are stained with DAPI (blue); scale bar=25μm.
